# Analysis of the mechanism of skeletal muscle atrophy from the pathway of decreased protein synthesis

**DOI:** 10.3389/fphys.2025.1533394

**Published:** 2025-04-25

**Authors:** Peng Chen, Fangfang Jia, Meng Wang, Shengbo Yang

**Affiliations:** Department of Anatomy, Zunyi Medical University, Zunyi, China

**Keywords:** skeletal muscle atrophy, protein synthesis, mechanism, pathway, mTOR

## Abstract

Skeletal muscle atrophy is associated with denervation, cancer, diabetes, aging, immobilization, and inflammation, which can significantly impair mobility. It is primarily attributable to increased protein catabolism alongside reduced protein synthesis, although the precise mechanisms underlying this process are not yet fully known. Unlike in the pathway driving increased catabolism, fewer studies have explored the mechanism underpinning muscle atrophy under reduced protein synthesis. Therefore, this study aimed to focus on summarizing relevant studies on the reduction of protein synthesis leading to skeletal muscle atrophy, as driven by alterations in pathways such as the insulin-like growth factor-1-phosphatidylinositol 3-kinase-protein kinase B-rapamycin signaling pathway, glycogen synthase kinase-3, glucocorticoids, 5′-adenosine monophosphate-activated protein kinase, branched-chain amino acid sensors, myostatin, long-term proinflammatory factors, oxidative stress and mitochondrial dysfunction, calciumion concentration, activating transcription factor 4, and glycyl-tRNA synthetase alterations. Consolidating these data will provide a foundation and theoretical basis for further investigation into the mechanisms of muscle atrophy from the perspective of reduced protein synthesis pathways.

## 1 Introduction

Skeletal muscle, the body’s largest protein storehouse and primary site of glucose metabolism, maintains the balance of protein synthesis and degradation under physiological conditions (Yin et al., 2021). Disruptions to this balance—due to denervation, cancer, diabetes, aging, immobilization, or inflammation—induce skeletal muscle atrophy, as evidenced by degradation of myofibrils, reduced muscle mass, decreased muscle fiber cross-sectional area, increased mitochondrial autophagy, and a net loss of cytoplasmic, cellular organelles, and total proteins (Cruz-Jentoft et al., 2019; Liang et al., 2024). Beyond significantly impairing mobility, skeletal muscle atrophy increases the risk of secondary fractures and even life-threatening injuries. Currently, the treatment of skeletal muscle atrophy remains a global challenge, despite extensive research on the mechanisms of muscle atrophy through increased proteolysis and decreased synthesis. Here, we review and analyze the relevant mechanisms of skeletal muscle atrophy from the relatively less-studied pathway of reduced protein synthesis to provide a foundation for further in-depth exploration of the mechanisms and prevention of skeletal muscle atrophy.

## 2 Overview of skeletal muscle atrophy mechanisms

The maintenance and regulation of skeletal muscle mass are mainly attributed to two processes: protein turnover and myonuclear turnover (Wilburn et al., 2021). During skeletal muscle atrophy, an imbalance in protein homeostasis can result from activated muscle protein degradation, inhibited muscle protein synthesis, reduced myonuclei accumulation, or increased myonuclei loss; these factors collectively contribute to a net loss of muscle proteins, eventually triggering muscle atrophy (Wilburn et al., 2021). There are four protein degradation pathways in muscular atrophy: ubiquitin-proteasome, cysteine aspartate protease, autophagy-lysosome, and calcineurin activation pathway. These four degradation pathways interact and often function in concert (Yin et al., 2021). In addition to increased protein degradation, a critical factor in skeletal muscle atrophy is decreased protein synthesis, regulated by pathways and molecules such as insulin-like growth factor-1-phosphatidylinositol 3-kinase-protein kinase B-mammalian target of rapamycin (IGF-1-PI3K-Akt-mTOR) pathway, glycogen synthase kinase 3 (GSK3), adenosine 5′-monophosphate (AMP)-activated protein kinase (AMPK), branched-chain amino acid sensors, glucocorticoids, myostatin (MSTN), and activating transcription factor 4 (ATF4).

## 3 Mechanisms of reduced protein synthesis leading to muscular atroph

### 3.1 Insulin/IGF-1-PI3K-Akt-mTOR pathway inhibition

Insulin and IGF-1 are potent anabolic factors that maintain muscle growth and regulate protein synthesis in skeletal muscle (Yoshida and Delafontaine, 2020). Changes in IGF-1 signaling affect the size and function of muscle fibers, inducing protein synthesis with inhibition of its catabolism and promoting muscle mass gain through activation of the PI3K/Akt/mTOR pathway (Yoshida and Delafontaine, 2020; Canfora et al., 2022; Baht et al., 2020).

**FIGURE 1:**
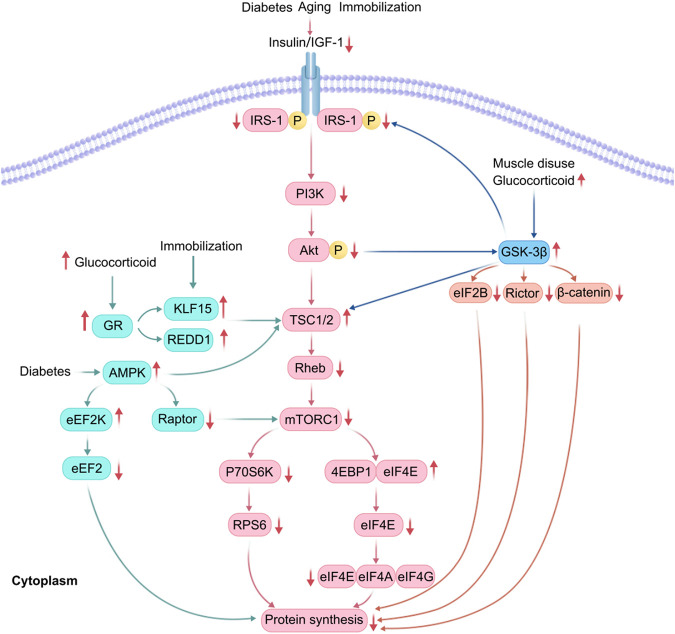
Adenosine 5′-monophosphate (AMP)-activated protein kinase (AMPK) and glycogen synthase kinase 3 (GSK3) β are involved in the reduction of protein synthesis. In patients with diabetes, glucocorticoid overflow induces insulin and insulin-like growth factor-1 (IGF-1) resistance, decreases the activity of protein kinase B (Akt), increases the expression of regulated in development and DNA damage response (REDD1) and Kruppel-like Factor 15 (KLF15), and activated AMPK, thereby blocking mammalian target of rapamycin complex 1 (mTORC1) signaling. GSK-3β is also activated under muscle disuse and glucocorticoid overflow, thus enhancing the phosphorylation of EIF2B, Rictor, and β-catenin, consequently inhibiting protein synthesis.

Insulin and IGF-1 are activated by binding to the insulin receptor on the plasma membrane of target cells, initiating a downstream signaling cascade that results in phosphorylation and activation of insulin receptor substrate 1 (IRS-1), PI3K, and Akt phosphorylation (Yoshida and Delafontaine, 2020; Sharlo et al., 2021; White, 2021). Akt activation phosphorylates and inactivates tuberous sclerosis complex 2 (TSC2), which promotes the activation of mammalian target of rapamycin complex 1 (mTORC1) by Ras homolog enriched in brain (Rheb), 70 kDa ribosomal protein S6 kinase (p70S6K) phosphorylation, and ribosomal protein S6, consequently promoting protein synthesis (Ji et al., 2022). Additionally, mTORC1 phosphorylates eukaryotic translation initiation factor 4E binding protein (4EBP1), releasing it from eukaryotic translation initiation factor 4E (EIF4E). Free EIF4E can bind with EIF4A and EIF4G to form a translation initiation complex, which initiates the binding of mRNAs to ribosomes, promotes translation initiation, and increases protein synthesis (Ji et al., 2022; Grüner et al., 2016) (Figure 1).

However, following hind limb suspension in rats, muscle ribosome degradation increases, biosynthesis decreases, and translational capacity diminishes (Figueiredo et al., 2021; Mathis et al., 2017). Furthermore, inhibition of mTORC1 causes a decrease in ribosomal DNA transcription, promotes ribose autophagy (Figueiredo et al., 2021), and rapidly dephosphorylates 4EBP1 and p70S6K, inhibiting the PI3K/Akt/mTOR pathway (Figueiredo et al., 2021). Additionally, E3 ubiquitin ligase Casitas B-cell lymphoma-b protein leads to diminished protein synthesis due to attenuated PI3K-dependent signaling and Akt dephosphorylation (Sharlo et al., 2021) (Figure 1). Another study has shown that Kr ü ppel like factor 15 (KLF15) and IL-6 are upregulated in skeletal muscle of mice with limb fixation. If KLF15 is deficient or IL-6 is systemically deficient, mice will be protected from fixation induced muscle atrophy (Hirata et al., 2022).

### 3.2 GSK3 activation

GSK3 is a serine/threonine kinase that regulates cytoskeletal composition and protein synthesis during the growth and development of the organism (Hajka et al., 2021). It has two isoforms, GSK3α and GSK3β, activated by autophosphorylation of tyrosine 279 and tyrosine 216, respectively (Patel and Werstuck, 2021). In response to insulin stimulation, Akt can lead to a decrease in GSK3α/β activity by phosphorylating serine 21 of GSK3α and serine 9 of GSK3β (Patel and Werstuck, 2021).

The dopaminergic D2 receptor activation promotes complex formation between Akt, GSK3, β-arrestin, and protein phosphatase 2A (PP2A). PP2A promotes dephosphorylation, which inactivates Akt and activates GSK3α at serine 21 and GSK3β at serine 9. GSK3 then facilitates complex formation, further activating GSK3α/β, inhibiting the PI3K/Akt/mTORC1 signaling pathway and reducing protein synthesis (Patel and Werstuck, 2021; Beurel et al., 2015; Papadopoli et al., 2021).

**FIGURE 2:**
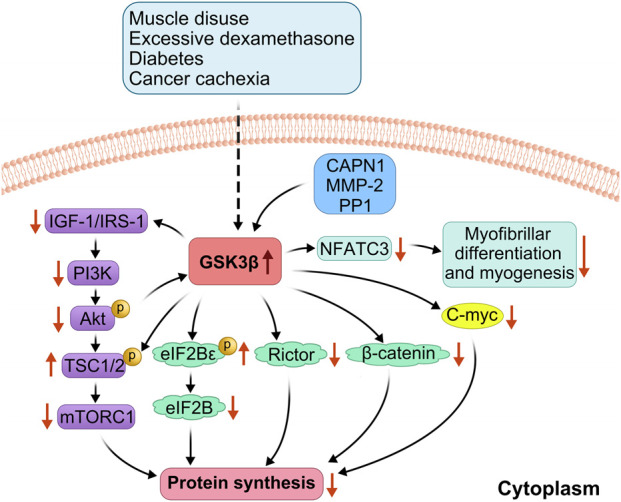
Detailed mechanism of GSK3β involvement in reduced protein synthesis. GSK-3β is activated during glucocorticoid overflow and muscle disuse, enhancing phosphorylation of EIF2B, Rictor and β-catenin, and also directly phosphorylates the Thr 58 site of cmy-c, leading to inhibition of the protein translation process; calpain-1(CAPN1), matrix metalloproteinase-2 (MMP2) and PP1 dephosphorylated the GSK3β, leading to GSK-3β activation, causing serine dephosphorylation of insulin-like growth factor 1 receptor and IRS-1 and inhibition of the IGF-1/PI3K/Akt/mTORC1 signaling pathway, which reduces protein synthesis; and inhibition of nuclear factor of activated T-cells c3(NFATc3), which inhibits myocyte differentiation and myogenesis.

GSK3β isoforms are predominant in reducing protein synthesis (Hajka et al., 2021) through multiple mechanisms (Figure 1) (Figure 2):

(1) In cases of rat soleus muscle atrophy induced by dexamethasone and muscle disuse, dephosphorylation of GSK3β-Ser9 increases GSK3β activity, inducing TSC2 phosphorylation and subsequent inhibition of mTORC1, which reduces protein synthesis (Mirzoev et al., 2021);
(2) GSK3β phosphorylates the ε subunit of eukaryotic initiation factor 2B, negatively regulating mRNA translation initiation (Beurel et al., 2015);
(3) GSK3β phosphorylates β-catenin, preventing its nuclear translocation and promoting its proteasomal degradation, thereby decreasing translational capacity (Beurel et al., 2015);
(4) Dephosphorylation of GSK3β by calpain-1 leads to phosphorylation at the threonine 58 site of the cytosolic myeloblastoma virus oncogene, causing ubiquitination and proteasomal degradation, which inhibits protein translation (Mirzoev et al., 2021; Mirzoev, 2020);
(5) Dephosphorylation of serine 9 by calpain-1, matrix metalloproteinase-2, and PP1, also results in the serine dephosphorylation of insulin-like growth factor 1 receptor and IRS-1, thereby inhibiting the IGF-1/PI3K/Akt/mTORC1 signaling pathway (Mirzoev et al., 2021);
(6) Phosphorylation of rapamycin-insensitive mTOR chaperone proteins leads to inactivation of mTORC2, which inhibits Akt and the Akt-mTORC2 pathway (Wang et al., 2022);
(7) Inhibiting nuclear localization of the T cell transcription factor nuclear factor c3 and gene transcription mediated by this factor inhibits myoblast differentiation and generation (van der Velden et al., 2008).

### 3.3 Glucocorticoid overflow

Endogenous and exogenous glucocorticoid spillage can negatively affect skeletal muscle mass and function (Britto et al., 2014). Specifically, when glucocorticoids are present in excess, their receptors promote the upregulation of regulated in development and DNA damage response 1 (REDD1) and Kruppel-like Factor 15 (KLF15) (Britto et al., 2014). Upregulated REDD1 and KLF15 activate their upstream target, TSC2, inhibiting mTORC1. Upregulated KLF15 promotes branched-chain amino acid catabolism mediated by branched-chain transcarbamylase 2 with reduced mTORC1 activity (Nunes et al., 2022). Under diabetic conditions, downregulation of E3 ubiquitin ligase WWP1 and upregulation of KLF15 lead to muscle atrophy (Hirata et al., 2019). Glucocorticoids also induce insulin and IGF-1 resistance, upregulate MSTN expression, and culminate in reduced protein synthesis (Ji et al., 2022; Franco-Romero and Sandri, 2021) (Figure 1).

### 3.4 Activation of AMPK

AMPK is a negative regulator of protein synthesis in skeletal muscle and is activated in the context of diabetes or triggered insulin resistance (such as glucocorticoid therapy, chronic inflammation, and lipid overload) (Franco-Romero and Sandri, 2021). Inhibition of the mTORC1 signaling pathway leads to reduced protein synthesis in skeletal muscle mainly through four pathways (White, 2021):

(1) Phosphorylation of mTOR-Thr2446, which prevents Akt-mediated phosphorylation of mTOR-Ser2448, thereby inhibiting translation initiation (Franco-Romero and Sandri, 2021);
(2) Phosphorylation and activation of TSC2, which inhibits mTORC1 signaling (Papadopoli et al., 2021);
(3) Direct phosphorylation of regulatory associated protein of mTOR (RAPTOR), which promotes the chelation of RAPTOR by 14-3-3 proteins, inhibits downstream p70S6K and 4EBP1 signaling by RAPTOR, and ultimately impairs mTORC1 activity, reducing mRNA translation, leading to reduced protein synthesis (White, 2021; Mirzoev, 2020);
(4) AMPK can directly phosphorylate and activate eukaryotic elongation factor 2 kinase (eEF2K), leading to phosphorylation and inactivation of eEF2 at the Thr56 site. This inhibits the binding of elongation factors to ribosomes, resulting in decreased translation efficiency and reduced protein synthesis (Thomson, 2018) (Figure 1).

### 3.5 Increased expression of branched-chain amino acid sensors

**FIGURE 3:**
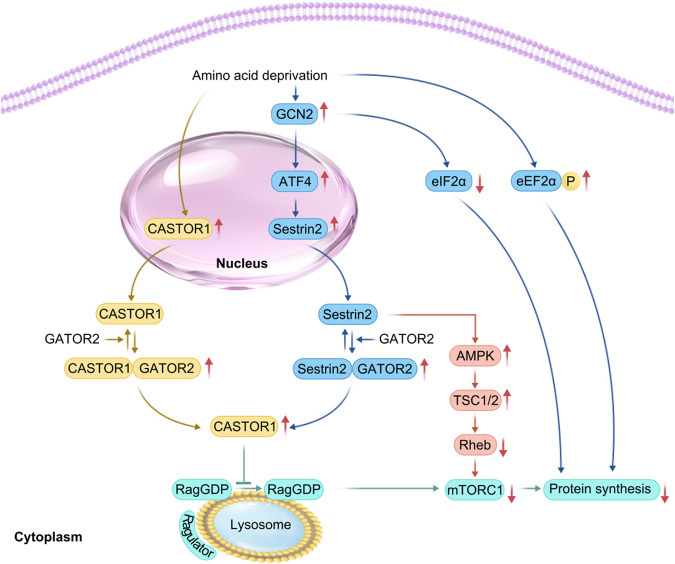
Related mechanisms of amino acid deprivation leading to skeletal muscle atrophy. In the case of amino acid deprivation, (CASTOR1) expression is increased, whereas GCN2 is activated. This promotes activating transcription factor 4 (ATF4), upregulation, increasing Sestrin2 levels downstream of ATF4 in response to cell stress, thereby promoting the formation of Sestrin2-Gator2 and Castor1-Gator2 complex. Once free GATOR1 is increased, mTORC1 signaling is inhibited, thereby yielding a reduction in protein synthesis. The increased phosphorylation of EIF2α and eEF2α impedes the initiation of translation and subsequent elongation, resulting in reduced translational efficiency.

Branched-chain amino acids, especially leucine, promote muscle protein synthesis and prevent catabolism by activating the mTOR signaling pathway. They can also serve as substrates for protein synthesis (Dimou et al., 2022). However, an increase in the expression of branched-chain amino acid sensors inhibits mTORC1 activity. For example, selenocysteine 2 protein (Sestrin2) is a leucine sensor that negatively regulates the mTORC1 pathway (Chantranupong et al., 2014). Sestrin2, leucine, and GTPase activating protein activity toward rags complex 2 (GATOR2) maintain an equilibrium (Wolfson and Sabatini, 2017). Under conditions of leucine deprivation or DNA damage, general control nonderepressible kinase 2 (GCN2) is activated, and ATF4 is upregulated, inducing upregulation of Sestrin2 expression in response to cellular stress, increased binding of Sestrin2 to GATOR2, disruption of the interaction of GATOR2 with GATOR1. Consequently, this increases free GATOR1 levels, enhances the interaction of Sestrin2 with Ras-related GTPase (Rag GTPase), inactivates Rag GTPase, and recruits TSC2 to the lysosome to inactivate Rheb. This process blocks leucine-induced lysosomal localization of mTORC1 and inhibits its kinase activity, ultimately decreasing the phosphorylation of p70S6K and 4EBP1, thereby inhibiting the mTORC1 signaling pathway (Wolfson and Sabatini, 2017; Ye et al., 2015; Budanov and Karin, 2008). Activated general control nonderepressible kinase 2 (GCN2) also phosphorylates eukaryotic initiation factor 2α (EIF2α), resulting in blocked GDP to GTP conversion on EIF2 and delayed translation initiation (Lehman et al., 2015). Leucine deprivation also increases the phosphorylation of eEF2α and slows the elongation rate, decreasing translation efficiency (Lehman et al., 2015). In leucine deficiency, Sestrin2 activates AMPK through direct interaction and induces phosphorylation of TSC2, which converts Rheb from a GTP-bound state to a GDP-bound state, inactivating Rheb and thus inhibiting the mTORC1 pathway (Xu et al., 2019). Under arginine depletion conditions, the cytosolic arginine sensor for mTORC1 subunit 1 (CASTOR1) can bind to GATOR2, disrupting the interaction between GATOR2 and GTPase activating protein activity toward rags complex 2 (GATOR1). This increase in free GATOR1 promotes the conversion of Rag GTPase to Rag GDPase, preventing the activation of the mTORC1 pathway (Franco-Romero and Sandri, 2021) (Figure 3).

### 3.6 Upregulation of MSTN expression

**FIGURE 4:**
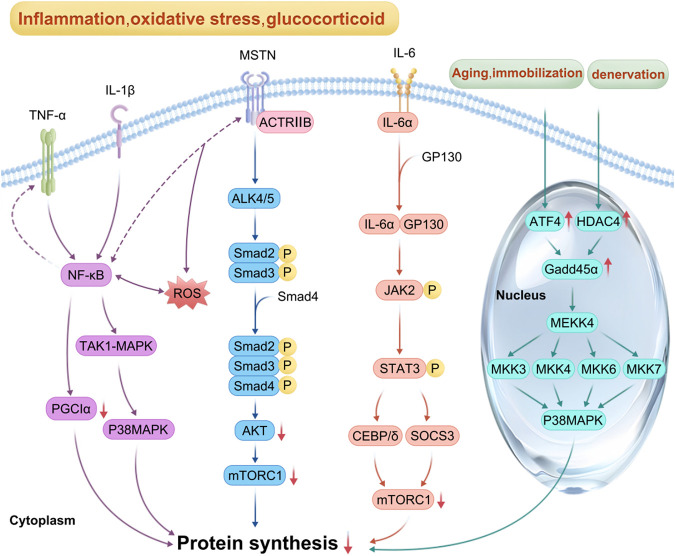
Mechanism of inflammatory factors, myostatin (MSTN) and ATF4 involved in reduced protein synthesis: In the context of inflammation, oxidative stress and glucocorticoid overflow, pro-inflammatory cytokines such as IL-1β, IL-6, and TNF-α activate NF-κB, p38MAPK, and JAK-STAT pathways. The activation of NF-κB pathway also promotes the expression of TNF-α and MSTN, whereas the increase of ROS also activates NF-κB pathway and inhibits mTORC1 signal transduction; MSTN first combines with actriib, and then with ALK4/5 to synthesize a complex and activate it. Smad2/3 phosphorylation induced Smad2/3 and Smad4 to form a complex, leading to the inactivation of Akt. Once Akt/mTORC1 signaling was blocked, protein synthesis decreased. In the process of fasting, immobilization, and aging, activated ATF4 promotes the expression of GADD45α protein, whereas, under denervation, histone deacetylase 4 (HDAC4) promotes the expression of GADD45α. Briefly, these factors interact to inhibit mTORC1 signal transduction, resulting in reduced protein synthesis, which eventually leads to muscle atrophy.

MSTN, a transforming growth factor-β superfamily member, is produced and secreted mainly by skeletal muscle. It inhibits the proliferation and differentiation of myoblasts and is a negative regulator of skeletal muscle growth and development (Yoshida and Delafontaine, 2020; Chen et al., 2021). MSTN synthesis is increased by stimuli such as inflammation, oxidative stress, angiotensin II, and glucocorticoids (Ji et al., 2022). On the surface of myocytes, MSTN binds first to Activin Receptor Type IIB and then to Activin Receptor Like Kinase 4/5 (ALK4/5) to form a complex and activate it. Subsequently, phosphorylation of Smad2/3 induces the formation of a complex between Smad2/3 and Smad4, inactivation of Akt, blockage of Akt/mTORC1 signaling, and reduced protein synthesis (Sartori et al., 2021; Verzola et al., 2019). MSTN signaling can also be independent of Smad regulation (Chen et al., 2021). Activation of the nuclear transcription factor-κB (NF-κB) pathway can be induced by tumor necrosis factor-α (TNF-α), which stimulates the upregulation of MSTN expression in myoblasts and inhibits the proliferation of myoblasts through the activation of p38 mitogen-activated protein kinase (p38 MAPK) via the transforming growth factor beta-activated kinase 1-mitogen-activated protein kinase 6 (TAK1-MAPK6) pathway (Chen et al., 2021). In patients with chronic obstructive pulmonary emphysema, MSTN is elevated in skeletal muscle, which activates the NF-κB pathway, induces elevated reactive oxygen species (ROS) in skeletal muscle via the Smad2/3-TNF-α-NF-κB pathway, and inhibits the Akt/mTORC1 pathway, leading to decreased protein synthesis (Ji et al., 2022) (Figure 4).

### 3.7 Chronic pro-inflammatory factor stimulation

In sepsis, cancer, chronic heart failure, and diabetes, high levels of pro-inflammatory cytokines such as IL-1β, IL-6, and TNF-α act directly on NF-κB, p38 mitogen-activated protein kinase, and Janus kinase-Signal Transducer and Activator of Transcription (JAK-STAT) pathways through the corresponding receptors, resulting in increased expression of suppressor of cytokine signaling 3, degradation of IRS1, and inhibition of the Akt/mTORC1 pathway, and decreased protein synthesis (Ji et al., 2022; Xu et al., 2019). During sepsis, activated IL-6 binds to cell surface α-receptors, leading to Glycoprotein 130 (GP130) recruitment and homodimerization, and the activated IL-6-α receptor binds to GP130 to form a complex, leading to GP130 phosphorylation and tyrosine kinase of the JAK family (mainly JAK1, JAK2, and tyrosine kinase 2) activation and binding to STAT, which phosphorylates STAT, leading to enhanced expression of CCAAT enhancer-binding protein δ, interference with insulin-induced IRS-1/Akt signaling, and inhibition of protein synthesis (Ji et al., 2022; Zanders et al., 2022) (Figure 4).

### 3.8 Oxidative stress and mitochondrial dysfunction

Oxidative stress will produce ROS, which is mainly produced in mitochondria, and accumulation of ROS can induce muscle atrophy under a variety of pathological conditions (such as aging, disuse, diabetes, denervation and cancer cachexia) (Xu et al., 2025; Eshima et al., 2025; Eshima et al., 2023). When the mitochondria of skeletal muscle are damaged due to ROS production, lipid peroxidation within the mitochondria intensifies, leading to increased mitochondrial damage (Xu et al., 2025; Hughes et al., 2022).

During the aging process, high levels of ROS induce oxidative damage to nucleic acids, proteins, and lipids, leading to a decrease in protein synthesis (Xu et al., 2025). Under high oxidative stress conditions, the activity or content of dihydropyridine receptors in muscles is significantly reduced, the expression of dihydropyridine receptors is decreased, the number of unconjugated ryanodine receptors is increased, the release of Ca^2+^in the sarcoplasmic reticulum is reduced, resulting in a significant decrease in the excitation contraction coupling of muscles, a decrease in muscle strength, and ultimately leading to muscle atrophy (Xu et al., 2025; Ahn et al., 2022).

Limb disuse and diabetes will increase the oxidative status of muscle tissue, reduce the level of antioxidant enzymes in the body, lead to excessive increase of ROS, reduce mitochondrial biology, and promote oxidative damage (Chen et al., 2023; Lv et al., 2022). When the load on muscles increases, neuronal nitric oxide synthase (nNOS) is activated, and nitric oxide reacts with superoxide to produce peroxynitrite, activating the transient receptor potential cation channel, subfamily V, member 1, inducing Ca^2+^ release in the sarcoplasmic reticulum, triggering mTOR activation, and inducing increased protein synthesis (Ito et al., 2013). On the contrary, disuse of limbs leads to a decrease in protein synthesis. Elevated ROS levels in patients with diabetes can directly damage β cells in pancreatic islets and promote apoptosis, induce insulin resistance, inhibit the PI3K/Akt/mTORC1 pathway, impede the initial stages of mRNA translation, and reduce protein synthesis (Zhang et al., 2023; Kubat et al., 2023). It is worth mentioning that deuterium-enhanced polyunsaturated fatty acids can resist the lipid peroxidation chain reaction triggered by ROS, inhibit lipid peroxidation, and prevent muscle atrophy and weakness caused by diabetes (Eshima et al., 2025).

In disuse muscular atrophy induced by denervation, skeletal muscle blood perfusion is reduced, resulting in ischemia and hypoxia. Long-term ischemia and hypoxia promote excessive production of ROS, resulting in oxidative damage, increase of inflammatory factors, activation of inflammatory response, and aggravation of skeletal muscle injury (Chen et al., 2023; Zhang et al., 2023).

In cancer cachexia patients, the expression of IL-6, TNF-α, and MSTN increases, leading to activation of the STAT3 and NF-κB pathways, increased inflammation, decreased activity and expression of peroxisome proliferator activated receptor-γ coactivator factor-1α, decreased antioxidant capacity, increased oxidative stress, reduced mitochondrial biogenesis, resulting in inhibition of the synthetic metabolic pathway (Abu et al., 2023; Carson et al., 2016) (Figure 4).

### 3.9 ATF4 activation

ATF4, a member of the CREB/ATF family, participates in anabolic cellular stress responses and acts as a downstream mediator of the EIF2α kinase and mTORC1 in response to anti-insulin/IGF-1 signaling. It is activated during oxidative stress and endoplasmic reticulum (ER) stress (Ebert et al., 2019). In patients with lateral sclerosis, unfolded and misfolded proteins accumulate in the lumen of the ER. Immunoglobulin-binding proteins bind to these proteins, activating the protein kinase RNA-like ER kinase, which leads to increased phosphorylation of EIF2α and the expression of ATF4 mRNA while inhibiting EIF2B activity. This process reduces ribosome assembly and protein synthesis (Sartori et al., 2021; Chen et al., 2015; Ebert et al., 2022). During fasting, immobilization, and aging, activated ATF4 promotes growth arrest and DNA damage-induced 45α (Gadd45α) protein expression. This protein binds to mitogen-activated protein kinase kinase kinase 4 (MAP3K4) to form a complex that activates downstream mitogen-activated protein kinases 3, 4, 6, and 7, leading to p38MAPK activation, which reduces mitochondrial production, inhibits protein synthesis, and promotes muscle atrophy (Ebert et al., 2019). In contrast, during denervation, growth arrest and DNA damage-induced 45α (Gadd45α) protein expression is induced by histone deacetylase 4 (Bongers et al., 2013; Bullard et al., 2016) (Figure 3).

### 3.10 Downregulation or mutation of glycyl-tRNA synthetase expression

Glycyl-tRNA synthetase (GARS) mediates protein synthesis by catalyzing the binding of glycine to tRNA to generate glycyl-tRNA, generating glycine residues to cognate tRNAs during mitochondrial and cytoplasmic protein translation (Galindo-Feria et al., 2022). GARS mutations can lead to type 2D Charcot Marie Tooth disease (Zhao et al., 2021). These mutations cause ribosomal arrest by inhibiting the regulation of glycyl-tRNA at the ribosomal A site, which induces phosphorylation of EIF2α and activates the integrated stress response, leading to inhibition of translational initiation (Mendonsa et al., 2021). The downregulation or complete deletion of GARS expression leads to mitochondrial translational defects in neuronal and myogenic cells, causing reduced protein synthesis (Wang et al., 2016; Zhang et al., 2021).

## 4 Summary and outlook

Muscle atrophy is characterized by the inhibition of protein synthesis, resistance to anabolic stimuli, and protein degradation due to many factors. While intensive research on protein synthesis has focused on the signaling mechanisms associated with the mTORC1 pathway, which involves the regulation of changes in factors such as glycogen synthase kinase-3, glucocorticoids, 5′-AMP-activated protein kinase, branched-chain amino acid sensors, myostatin, long-term proinflammatory factors, oxidative stress and mitochondrial dysfunction, calciumion concentration, activating transcription factor 4, and glycyl-tRNA synthetase (Table 1). Currently, it is known that inhibition of mTORC1 can cause a decrease in ribosomal DNA transcription and promote riboautophagy. However, the mechanisms underlying the biological decline of ribosomes and mitochondria, as well as the relationship between post-translational modifications of proteins and decreased protein synthesis, are still unclear. In the context of denervation, cancer cachexia, diabetes mellitus, aging, oxidative stress, immobilization, and inflammation, it is necessary to determine whether all regulatory mechanisms leading to decreased protein synthesis are present in each muscle atrophy disease. These investigations may indicate that relying on a single gene or pathway to study the underlying mechanisms is insufficient. Instead, verifying the commonality of these reduced protein synthesis factors in various myasthenic diseases is essential to decode the molecular mechanism of skeletal muscle atrophy.

**TABLE 1:** Factors related to decreased protein synthesi.

Influencing factors	Key molecules or pathways	Mechanism	Causes/diseases	References
IGF-1/PI3K/Akt/mTOR pathway inhibition	IGF-1 Akt mTORC1	Inhibition of ribosome biosynthesis and protein translation initiation Reduction in protein synthesis	Malnutrition, Insulin resistance Aging Immobilization	Figueiredo et al. (2021), Mathis et al. (2017)
GSK3β activation	TSC2 mTORC1 eIF2Bε β-catenin	Phosphorylation of TSC2 and inhibition of mTORC1 Phosphorylation of eIF2Bε and inhibition of translation initiation Phosphorylation of β-catenin and c-myc Serine dephosphorylation of IGF-1R and IRS-1	Excessive dexamethasone Muscle disuse	Mirzoev et al. (2021), Mirzoev (2020)
Glucocorticoid overflow	Dexamethasone KLF15 Insulin and IGF-1	Upregulation of KLF15 and REDD1 expression Promotion of branched chain amino acid catabolism Resistance to insulin and IGF-1	Diabetes Long-term use of glucocorticoid	Ji et al. (2022), Hirata et al. (2022), Britto et al. (2014), Franco-Romero and Sandri (2021)
Activation of AMPK	RAPTOR eEF2K TSC2	Phosphorylation and activation of TSC2 Phosphorylation of RAPTOR and reduction of mTORC1 activity Phosphorylation and activation of eEF2K	Diabetes Insulin resistance Chronic inflammation	White (2021), Papadopoli et al. (2021), Mirzoev (2020), Franco-Romero and Sandri (2021), Thomson (2018)
Branched-chain amino acid sensors upregulated expression	Leucine Sestrin2 mTORC1 EIF2α and eEF2α	Leucine deprivation blocks mTORC1 lysosomal localization Phosphorylation of EIF2α Leucine deprivation increases eEF2α phosphorylation AMPK activated by Sestrin2	Leucine deprivation DNA damage Malnutrition	Wolfson and Sabatini (2017), Ye et al. (2015), Budanov and Karin (2008), Lehman et al. (2015), Xu et al. (2019)
Upregulation of MSTN expression	Smad2/3 pathway NF-κB pathway p38-MAPK pathway	Activation of Smad2/3 pathway and NF-κB pathway	Inflammation Oxidative stress Angiotensin II and Glucocorticoids	Ji et al. (2022), Chen et al. (2021)
Chronic proinflammatory factor stimulation	TNF-α IL-6 JAK/STAT/C/EBPδ pathways	Activation of JAK/STAT/C/EBPδ pathway IRS-1/Akt signaling pathway impaired by pro-inflammatory cytokines	Chronic heart failure Cancer cachexia Sepsis Diabetes	Ji et al. (2022), Chen et al. (2021)
Oxidative stress and mitochondrial dysfunction	ROS Mitochondria ATP	Oxidative stress leads to ROS accumulation Mitochondrial function disrupted by ROS	Aging and muscle disuse Diabetes and cancer cachexia Denervation	Ji et al. (2022), Xu et al. (2025), Chen et al. (2023), Lv et al. (2022), Zhang et al. (2023)
Dysregulation of Ca^2+^ homeostasis	nNOS Ca^2+^	Denervation Reduced activity of Ca²⁺ release channels	Aging Oxidative stress Immobilization	Xu et al. (2025), Ahn et al. (2022), Ito et al. (2013)
ATF4 activation	Protein kinase RNA like endoplasmic reticulum kinase HDAC4	Upregulation of Gadd45α expression activates p38MAPK and inhibits protein synthesis	Fasting Immobilization Aging Denervation	Ebert et al. (2019), Bongers et al. (2013), Bullard et al. (2016)
